# Spatiotemporal force and motion in collective cell migration

**DOI:** 10.1038/s41597-020-0540-5

**Published:** 2020-06-24

**Authors:** Aashrith Saraswathibhatla, Emmett E. Galles, Jacob Notbohm

**Affiliations:** Department of Engineering Physics, University of Wisconsin-Madison, Madison, WI USA

**Keywords:** Biological physics, Biomedical engineering, Collective cell migration, Cellular motility

## Abstract

Cells move in collective groups in biological processes such as wound healing, morphogenesis, and cancer metastasis. How active cell forces produce the motion in collective cell migration is still unclear. Many theoretical models have been introduced to elucidate the relationship between the cell’s active forces and different observations about the collective motion such as collective swirls, oscillations, and rearrangements. Though many models share the common feature of balancing forces in the cell layer, the specific relationships between force and motion vary among the different models, which can lead to different conclusions. Simultaneous experimental measurements of force and motion can aid in testing assumptions and predictions of the theoretical models. Here, we provide time-lapse images of cells in 1 mm circular islands, which are used to compute cell velocities, cell-substrate tractions, and monolayer stresses. Additional data are included from experiments that perturbed cell number density and actomyosin contractility. We expect this data set to be useful to researchers interested in force and motion in collective cell migration.

## Abstract (summary)

**Table:** 

Measurement(s)	force • Motion • MDCK cell • cell migration
Technology Type(s)	*in vitro* microscopy with digital image analysis • time-lapse microscopy
Factor Type(s)	cell number density • cytochalasin D • CN03

Machine-accessible metadata file describing the reported data: 10.6084/m9.figshare.12378218

## Background & Summary

Collective cell migration is ubiquitous in multicellular organisms playing a fundamental role in embryogenesis, wound healing, and metastasis of cancer^1–3^. The collective motion is driven by active forces generated within the cell; these forces are balanced by forces transmitted to the substrate and to other cells. The complicated balance of forces results in phenomena such as swirls, oscillations, and distinct changes between migratory and non-migratory phases^4–8^. These are emergent phenomena, describing the collective result of forces produced by the individual cells. As the emergent phenomena span scales from the individual cell to the collective, it is challenging to design experiments to reveal fundamental relationships between force and collective motion. Theoretical models offer a means to address this challenge, as they can connect forces at the scale of a cell to motion of the collective. To verify assumptions, test predictions, and interpret results of these models, experimental data are required.

There exist numerous physics-based theoretical models that relate force and motion in a cell layer^7,9–22^. The formulation of those models may differ—for example, models have treated the cell layer as a continuum, a set of points on a lattice, a collection of polygons, or a group of self-propelled particles—but the models often have similar components, namely forces within the cell layer and at the cell-substrate interface balance with a viscous friction caused by cell motion. However, the models differ in the specific relationships between force and motion. For example, some models define the actively generated force to have a direction that is persistent over a short time but otherwise random^15,16^, whereas other models give cells a tendency for the active force to align with the direction of cell velocity^7,9–11,14,19^ or the orientation of cell elongation^20^. Some models may also give cells a tendency to direct force so as to migrate away from cell-cell contacts or toward free space^7,13,14,17^. As the different assumptions made by the models could lead to different conclusions, experiments are needed to validate model assumptions and test model predictions. Furthermore, experiments can advance the models by making new observations not predicted by current models. For example, experimental observations that collective cell groups move in larger clusters as cell number density is increased^23–26^ later inspired theoretical models to investigate the effect of density on collective migration^10,11,14,19^. Additional examples of connections between theory and experiment are given in recent review articles^27–29^.

**Fig. 1:**
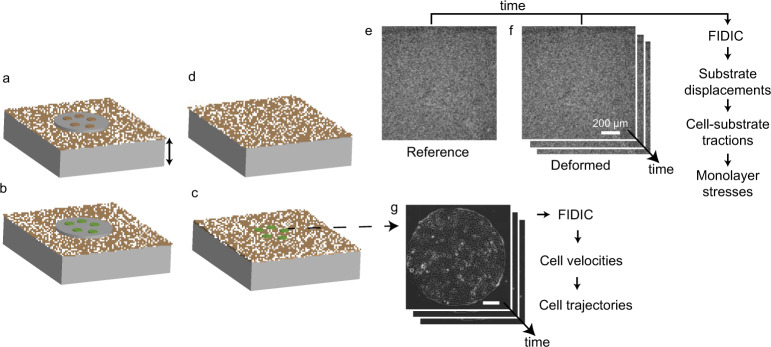
Experimental flowchart to measure cell velocities, cell-substrate tractions, and monolayer stresses. (**a**) A PDMS mask was placed on a polyacrylamide substrate embedded with fluorescent particles (see Methods), and collagen I was adhered to the substrate at locations of the holes in the mask. (**b**) Cells (green) were seeded to form cellular islands of desired diameter. (**c**) The PDMS mask was removed, and time-lapse imaging of a confluent cell island and the fluorescent particles was performed simultaneously. (**d**) The cell island was removed using 0.05% trypsin, which allowed the substrate to recover to a stress-free reference state used for computing cell-substrate tractions. (**e,f**) The reference image was correlated with the time-lapse images of the deformed substrate to obtain the substrate displacements. (**g**) Consecutive phase contrast images of the cell island were correlated to compute cell velocities.

**Fig. 2:**
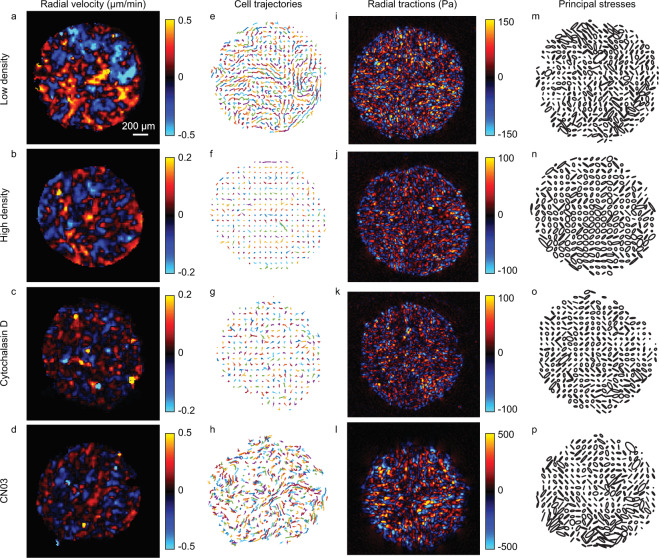
Representative plots showing velocities, trajectories, tractions, and stresses. (**a–d**) Radial component of cell velocities. (**e–h**) Cell trajectories. (**l–l**) Radial component of cell-substrate tractions. (**m–p**) Monolayer stress ellipses. Rows correspond to an island of low density (**a,e,i,m**), an island of high density (**b,f,j,n**), an island treated with cytochalasin D (**c,g,k,o**), and an island treated with CN03 (**d,h,l,p**).

**Fig. 3:**
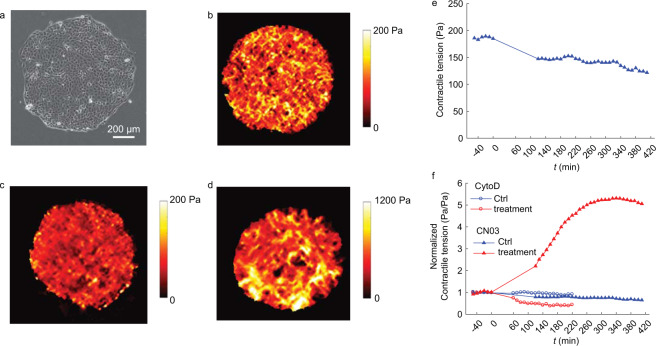
Technical validation of monolayer stresses. (**a**) Image of a representative cell island. (**b–d**) Colormaps of contractile tension for a control island (**b**), an island treated with cytochalasin D (**c**), and an island treated with CN03 (**d**). (**e**) Average contractile tension of a control island over time. Time *t* = 0 corresponds to the time that a treatment (in this case, vehicle control for CN03) is added. (**f**) Contractile tension is normalized by the average contractile tension for *t* < 0 and plotted over time. The data show representative results for cytochalasin D and CN03 along with their vehicle controls.

Despite the importance of experimental data of forces and motion in collective cell migration, there remains a lack of data sets that are easily accessible to the research community. To fill in this gap, we provide experimental data of collective cell migration in confined islands of 1 mm diameter^30^. These confined islands are one of multiple possible geometrical constraints, and the forces and motion may differ from other geometries such as larger cell layers and cell monolayers that are free to migrate outward, as in a wound healing assay. However, confinement has been considered in some theoretical models^7,10,21,22^, making this geometry a reasonable starting point. The data we provide include simultaneous measurements of cell velocities, cell-substrate tractions, and monolayer stresses. In addition, raw images collected during our experiments are available. The workflow of the experiments from patterning cell islands to time-lapse imaging to computing force and motion is shown in Fig. 1. Full details are available in the Methods section. Briefly, circular islands (1 mm diameter) were micropatterned onto polyacrylamide substrates having Young’s modulus 6 kPa. After cells were seeded to reach desired density, time-lapse imaging was performed, which provided the images required to compute cell velocities (Fig. 2), cell-substrate tractions (Fig. 2), and monolayer stresses (Figs. 2, 3) by employing digital image correlation, traction force microscopy, and monolayer stress microscopy^24,31–36^.

Experiments were performed for cell islands at low and high number density and for cell islands treated with an inhibitor and an activator of actomyosin contractility. These data are useful for studying scientific questions related to how density and cell forces affect the collective motion. The inhibitor of actomyosin contractility is cytochalasin D (0.05 μM), which was used at a concentration that reduces the tractions by 80%^8^. The activator for contractility is CN03 (2 µg/mL), which activates Rho, leading to an approximately 3-fold increase in cell tractions^8^. A summary of the data is in Table 1. For each set of data, we provide time-lapse images of cells and substrates embedded with fluorescent particles. Additionally, we provide computed cell displacements, tractions, and stresses. Overall, we expect the data to be useful for researchers to test and improve theoretical models and to refine methods for analysing the experimental data.

**Table 1:** Summary of experimental data.

System	No. of islands	Data name
Low density	4	low_density_islands
High density	4	high_density_islands
Cytochalasin D	6	cytoD_islands
CN03	8	cn03_islands

All experiments use islands of MDCK cells patterned on substrates having Young’s modulus of 6 kPa. Images, velocities, cell-substrate tractions, and monolayer stresses are provided for all experiments. Spatial resolution: 12 and 8 pixels (7.9 and 5.2 μm) for velocities and tractions/stresses respectively. Temporal resolution: 10 minutes.

## Methods

### Cell culture

The cells used were Madin-Darby canine kidney (MDCK) type II, transfected with green fluorescent protein attached to a nuclear localization signal. The cells were provided by members of David Weitz’s research group, Harvard University. The cells were maintained in low-glucose Dulbecco’s modified Eagle’s medium (Corning Cellgro 10-014-CV, Corning, NY) with 10% fetal bovine serum (FBS, Corning) and 1% G418 (Corning) in an incubator at 37 °C and 5% CO_2_. The cells were passaged every 2–3 days at 70–90% confluency. For the experiments at low density, high density, and using cytochalasin D, medium having 2% FBS was added 3–4 hours before the start of imaging and used for the duration of the experiments. For experiments with CN03, medium having 1% FBS was added one day before the experiment.

### Substrate fabrication

We followed the protocol described in our recent work^8^ to fabricate substrates of Young’s modulus 6 kPa with flurorescent particles for traction force microscopy. To begin, a polyacrylamide solution of 5.5% weight/volume (w/v) acrylamide (Biorad Laboratories, Hercules, CA), 0.2% w/v bisacrylamide (Biorad), 0.006% (w/v) ammonium persulfate (Biorad), and 0.002% (v/v) TEMED (Biorad) was prepared, and a 20 μL droplet was pipetted onto a #1.5 glass bottom dish (Cellvis, Mountain View, CA). A glass coverslip (18-mm diameter circle) was placed on the droplet to make a thin layer of gel, and the solution was allowed to polymerize at room temperature. A second polyacrylamide solution was prepared with the same composition as the first gel except 0.036% w/v fluorescent particles (diameter 0.5 µm, carboxylate-modified; Life Technologies) were added. The coverslip was removed from the gel and a 20 μL droplet of the second solution was pipetted onto the first gel. Again, a coverslip was placed on top. The dish was centrifuged upside down at 700 g for 10 minutes to localize the fluorescent particles onto the top of the second gel during polymerization. This method resulted in substrates having thickness in the range 100–120 μm.

### Micropatterning cell islands

Polydimethysiloxane (PDMS) (Sylgard 184, Dow Corning, Midland, MI) was prepared according to manufacturer instructions and poured into petri dishes at a thickness of 500 µm. The dishes were transferred to a hot plate to cure for 4 hours at 70 °C. The PDMS was cut into circles with a 16 mm punch, and 1 mm holes were made with a biopsy punch. Each resulting mask was sterilized in 70% ethanol and incubated in 2% Pluronic F-127 (Sigma-Aldrich, St. Louis, MO) to prevent cell adhesion to the PDMS. The masks were placed on the polyacrylamide substrates (Fig. 1a), and the open circular surfaces on the gel were functionalized with type I rat tail collagen (BD Biosciences, Franklin Lakes, NJ; 0.01 mg/mL, 1–2 mL per 18-mm diameter gel) using the crosslinker sulfo-SANPAH (Pierce Biotechnology, Waltham, MA; 2 mg in 200 mL 0.05 M HEPES). A solution of 500,000 MDCK cells per mL was prepared and 200–300 μL of the solution was pipetted onto each PDMS mask (Fig. 1b). 1–2 hours after seeding, the PDMS masks were removed and the glass bottom dishes were incubated at 37 °C and 5% CO_2_ for 12–16 hrs, during which time the cell islands reached the desired cell density.

### Time-lapse imaging

Dishes with desired cell density were placed in an H301 stage top incubator with UNO controller (Okolab USA Inc, San Bruno, CA) to maintain environmental conditions of 37 °C and 5% CO_2_. An Eclipse Ti-E microscope (Nikon, Melville, NY) with a 10 × 0.5 numerical aperture objective (Nikon) and an Orca Flash 4.0 digital camera (Hamamatsu, Bridgewater, NJ) running on Elements Ar software (Nikon) was used to image the cell islands and fluorescent particles in the substrates every 10 min (Fig. 1f,g). Low and high density islands were imaged every 10 min for 3 hr. Islands treated with cytochalasin D were imaged before the treatment every 10 min for 1 hr; they were then treated with cytochalasin D and imaged every 10 min from 60 to 220 min after the treatment. Islands treated with CN03 were imaged before the treatment every 10 min for 1 hr; they were then treated with CN03 and imaged every 10 min from 120 to 410 min after the treatment. The timing of imaging for the cytochalasin D and CN03 treatments differed, because CN03 took a longer time to affect cell tractions than cytochalasin D^8^. For both the cytochalasin D and CN03 treatments, half of the islands were treated with vehicle control, dimethyl sulfoxide (0.04 µM in 1 × phosphate buffered saline) and 1 × phosphate-buffered saline, respectively. After the time-lapse imaging, cell islands were incubated with 0.05% trypsin to release the cells from the substrate to obtain a traction-free reference image of the substrates (Fig. 1d,e).

### Cell velocities and trajectories

Using Fast Iterative Digital Image Correlation (FIDIC)^35^, consecutive time-lapse images of cell islands were correlated to extract cell motion over time using 48 × 48 pixel subsets at a spacing of 12 pixels (7.9 µm). Representative results of radial velocities are shown in Fig. 2a–d. Cell trajectories were computed using Matlab code called compute_cell_trajectories.m, and representative trajectories are shown in Fig. 2e–h.

### Traction force microscopy

Using FIDIC^35^, the time-lapse images of fluorescent particles were correlated with the reference image (Fig. 1e,f) to compute cell-induced substrate displacements. The displacements were computed using a 32 × 32 pixel subsets at a spacing of 8 pixels (5.2 µm). Cell-substrate tractions were computed using Fourier transform traction microscopy^31,36^. Figure 2i–l shows representative radial tractions applied by the cells.

### Monolayer stress microscopy

We employed monolayer stress microscopy^24,34^ which is based on force equilibrium between cell-substrate tractions and cell-cell stresses. Applying equilibrium in the two directions in the plane of the monolayer gives two equations,

1 $$\begin{array}{l}\frac{\partial {\sigma }_{xx}}{\partial x}+\frac{\partial {\sigma }_{xy}}{\partial y}+\frac{{T}_{x}}{h}=0\\ \frac{\partial {\sigma }_{xy}}{\partial x}+\frac{\partial {\sigma }_{yy}}{\partial y}+\frac{{T}_{y}}{h}=0\end{array}$$

where *T*_*i*_ are the components of the traction vector applied by the cell layer, *h* is the height of the monolayer, and *σ*_*ij*_ are the components of the in-plane stress tensor. As the stress tensor has three independent components, a third equation is required. The third equation comes from geometrical compatibility of deformations within the cell monolayer. Monolayer stress microscopy initially wrote compatibility of the strains^24,34^, and suggested stresses and strains to be related linearly, implying the monolayer to be an elastic solid. Here, we demonstrate that if a viscous fluid behaviour is assumed, the formulation is equivalent, and the computed stresses are the same. The formulation begins by writing compatibility of the strain rates:

2 $$\frac{{\partial }^{2}{\dot{\varepsilon }}_{xx}}{\partial {y}^{2}}+\frac{{\partial }^{2}{\dot{\varepsilon }}_{yy}}{\partial {x}^{2}}=2\frac{{\partial }^{2}{\dot{\varepsilon }}_{xy}}{\partial x\partial y}$$

where ${\dot{\varepsilon }}_{ij}$ are the components of the strain rate tensor. The simplest way to relate the strain rate to the stresses is to assume the cell monolayer to be a homogeneous, viscous fluid,

3 $${{\rm{\sigma }}}_{ij}=\left({K}_{1}-\frac{2}{3}{K}_{2}\right){\dot{\varepsilon }}_{kk}{{\rm{\delta }}}_{ij}+2{K}_{2}{\dot{\varepsilon }}_{ij}$$

where *K*_1_ and *K*_2_ are bulk and shear viscosities, respectively. The thermodynamic pressure is left out of Eq. 3, as it is unknown whether such a thermodynamic pressure exists in cells.

Stresses are calculated from Eqs. 1–3 using the method of finite elements using 2D plane stress elements. The finite element method requires at least three boundary conditions, which must be applied carefully. A thorough description of the effect of boundary conditions is given elsewhere^34^. In this data set, the cells are in islands which are in equilibrium. Thus, the boundary conditions should have no effect on the computed stresses and are only included to stabilize the computation. Three Dirichlet boundary conditions are applied to restrict motion at the leftmost node (no vertical motion) and rightmost node (no vertical or horizontal motion) of the monolayer to zero. To verify that the boundary conditions do not impact the results, we applied the boundary conditions to different nodes and observed the computed stresses to remain unchanged. After calculating the stresses *σ*_*ij*_, we computed the principal stresses *σ*_1_ and *σ*_2_, and plotted them as ellipses where the major axis of the ellipse is proportional to the magnitude of first principal stress, the minor axis is proportional to the magnitude of second principal stress, and the orientation of the ellipse gives the orientation of the first principal stress (Fig. 2m–p).

A difference between Eq. 3 and the original papers describing monolayer stress microscopy is that Eq. 3 applies for a viscous fluid whereas the original manuscripts introducing the method used equations for an elastic solid^24,34^. If, as in the original implementation of monolayer stress microscopy, Eq. 3 were written using strains rather than strain rates, it would represent the constitutive relationship for an elastic solid with constants *K*_1_ and *K*_2_ being moduli rather than viscosities. Here we chose to write the equations in terms of a fluid, because over long time scales the cell layer has no memory of its initial state and is thus better described as a viscous (or viscoelastic) fluid. Importantly, the distinction between elastic and viscous is unimportant, because the two implementations are equivalent, and give the exact same results, which can be understood as follows. The data input into monolayer stress microscopy are tractions, and the data output are stresses, both of which have the same units. Based on this fact, it may be reasonable to suspect that units of *K*_1_ and *K*_2_ do not affect the calculation. This reasoning is confirmed by considering the method used to solve for Eqs. 1–3. The equations are solved using the finite element method, which writes Eqs. 1–3 in the linear system *Ku* = *f*, where *K* is the stiffness matrix (which depends linearly on *K*_1_ and *K*_2_), *u* is a vector representing either displacements (for an elastic solid) or velocities (for a viscous fluid), and *f* is the force at each node, which is computed from the tractions. The method solves for the vector field *u* by computing *K*^−1^*f* and later computes stresses by multiplying by *K*. Because stresses are computed by multiplying by *K*^−1^ and again by *K*, the magnitude and units of *K* (and, therefore, *K*_1_ and *K*_2_) do not affect the computed stresses. The computed stresses do depend on a dimensionless ratio of *K*_1_ and *K*_2_, which for an elastic material is Poisson’s ratio. Dependence on this dimensionless ratio has been shown to be modest^34^. Therefore, this implementation of monolayer stress microscopy applies for either an elastic solid or a viscous fluid. By the correspondence principle of viscoelasticity, the method also applies for a viscoelastic material. Our implementation of monolayer stress microscopy is freely available (see section titled “Code availability”).

## Data Records

The time-lapse images, cell velocities, cell tractions, and intercellular stresses are uploaded to the data repository figshare (10.6084/m9.figshare.c.4945206)^30^. There are five folders: low_density_islands, high_density_islands, cytoD_islands, cn03_islands_1-4, and cn03_islands_5-8. Every folder contains subfolders corresponding to different cell islands. Each subfolder has time-lapse images of fluorescent particles (c1_island#.tif), cells (c2_island#.tif), a reference image of the fluorescent particles (c1_tryp_island#.tif) and cell island boundaries (domain.tif) which were detected using software based on ref. ^37^. The subfolders also have data files in Matlab format for cell displacements (cell_displacements.mat), cell-substrate tractions (tract_results.mat), and monolayer stresses (stress_results.mat).

In the folder cytoD_islands, islands 1–3 were treated with vehicle control, and islands 4–6 were treated with cytochalasin D. In the files tract_results.mat and stress_results.mat, the first 6 time points were before adding the cytochalasin D, and the remaining time points correspond to 60–220 min after the treatment. In cell_displacements.mat, all time points correspond to 60–220 min after adding cytochalasin D.

In the folder cn03_islands_1-4, islands 1-4 were treated with vehicle control, and in cn03_islands_5-8, islands 5–8 were treated with CN03. In the files tract_results.mat and stress_results.mat, the first 6 time points were before adding the CN03, and the remaining time points corresponded to 120–410 min after the treatment. In cell_displacements.mat, all time points correspond to 120–410 min after adding CN03.

## Technical Validation

Since the FIDIC algorithm has been rigorously validated by Bar-Kochba *et al*.^35^ on synthetic images of known displacements, no further technical validation of the FIDIC algorithm was performed. We validated results from traction force microscopy by analysing tractions in two different ways. Firstly, there should be zero tractions in regions having no cells. This requirement is confirmed in Fig. 2i–l, where tractions are large inside the cell islands and zero outside the cell islands. Quantification of the ratio of root-mean-square of traction in the regions with and without cells for a representative cell island (island01 of the low density data set) gives a value of 0.03, indicating negligible tractions outside the cell island. This comparison demonstrates that the traction measurements are free of systematic errors and have negligible noise. We recommend that other researchers use this same method of validation of traction data. Secondly, validation was performed based on a recently observed phenomenon wherein cells at the free edge of a monolayer tend to apply traction so as to pull themselves toward the free space^38^. Consistent with this observation, our experimental data show that the cells apply radially inward (negative) tractions, as shown in Fig. 2i–l.

The method of monolayer stress microscopy has been carefully considered in previous studies^24,34^ and validated by a particle-based theoretical model^14^. Validation of the data was performed before and after applying monolayer stress microscopy. Because monolayer stress microscopy is based on the principle of equilibrium, it is important that the measured tractions of the cell island be in force and moment equilibrium. A script was written to verify equilibrium and make minor correction to account for lack of equilibrium resulting from experimental error in the traction data. Representative monolayer stresses are in Fig. 3. The data were validated by inspecting a plot showing the average of the principal stresses, which we refer to as the contractile tension (Fig. 3b–d). The plot shows that the contractile tension varies smoothly over space with no jumps or discontinuities, which is consistent with previously published data^24,34^. Additional validation is performed by following the contractile tension over time, which also varies smoothly (Fig. 3e). A final validation is given by analysing the measured stresses in response to cytochalasin D (which decreases contractility) and CN03 (which increases contractility). As expected, the treatments decreased and increased, respectively, the contractile tension within monolayer (Fig. 3b–d,f), thus validating the data set.

## Usage Notes

The data sets are available in two formats—unprocessed images captured during the experiments and processed data giving cell velocities, tractions, and stresses. The raw images are expected to be of interest to researchers developing new methods for image analysis, traction force microscopy, or computing monolayer stresses. For example, it may be useful to compare displacements computed with FIDIC^35^ with results from new, improved image correlation algorithms.

The processed data will be useful to researchers interested in relating force and motion in collective migration. Theoreticians in particular may find these data useful for comparison to their models.

Examples of code for plotting the data are available in the scripts plot_displ_tractions.m, plot_stress.m, plot_cellvel.m, and compute_cell_trajectories.m available at https://github.com/jknotbohm/Cell-Traction-Stress-Velocity-Plots. These scripts offer a starting point for data analysis, but it is likely that they will have to be modified or rewritten by the user to address a specific question of interest.

## Data Availability

Digital image correlation is performed with Fast Iterative Digital Image Correlation (FIDIC) software^35^ written in Matlab and available from https://github.com/FranckLab/ or https://github.com/jknotbohm/FIDIC. Code to compute cell boundaries, cell-substrate tractions, and monolayer stresses is available at https://github.com/jknotbohm/Cell-Traction-Stress. One script in the software package to compute tractions is from https://github.com/CellMicroMechanics/Easy-to-use_TFM_package (ref. ^36^). Code to compute cell trajectories and representative code to plot results are available from https://github.com/jknotbohm/Cell-Traction-Stress-Velocity-Plots.
